# eIF2B is a decameric guanine nucleotide exchange factor with a γ_2_ε_2_ tetrameric core

**DOI:** 10.1038/ncomms4902

**Published:** 2014-05-23

**Authors:** Yuliya Gordiyenko, Carla Schmidt, Martin D. Jennings, Dijana Matak-Vinkovic, Graham D. Pavitt, Carol V. Robinson

**Affiliations:** 1 Department of Chemistry, Physical and Theoretical Chemistry Laboratory, University of Oxford, South Parks Road, Oxford OX1 3QZ, UK; 2 MRC Laboratory of Molecular Biology, University of Cambridge, Francis Crick Avenue, Cambridge CB2 0QH, UK; 3 Faculty of Life Sciences, Michael Smith Building, University of Manchester, Oxford Road, Manchester M13 9PT, UK; 4 Department of Chemistry, University of Cambridge, Lensfield Road, Cambridge CB2 1EW, UK; 5 These authors contributed equally to the work

## Abstract

eIF2B facilitates and controls protein synthesis in eukaryotes by mediating guanine nucleotide exchange on its partner eIF2. We combined mass spectrometry (MS) with chemical cross-linking, surface accessibility measurements and homology modelling to define subunit stoichiometry and interactions within eIF2B and eIF2. Although it is generally accepted that eIF2B is a pentamer of five non-identical subunits (α–ε), here we show that eIF2B is a decamer. MS and cross-linking of eIF2B complexes allows us to propose a model for the subunit arrangements within eIF2B where the subunit assembly occurs through catalytic γ- and ε-subunits, with regulatory subunits arranged in asymmetric trimers associated with the core. Cross-links between eIF2 and eIF2B allow modelling of interactions that contribute to nucleotide exchange and its control by eIF2 phosphorylation. Finally, we identify that GTP binds to eIF2Bγ, prompting us to propose a multi-step mechanism for nucleotide exchange.

## Abstract (web summary)

Eukaryotic Initiation Factor 2 (eIF2) initiates protein synthesis aided by its partner eIF2B, which stimulates guanine nucleotide exchange on eIF2. Here, Gordiyenko *et al*. show that eIF2B exists as a decamer and propose a model for its subunit arrangement that provides new insight into its function.

## Graphical abstract

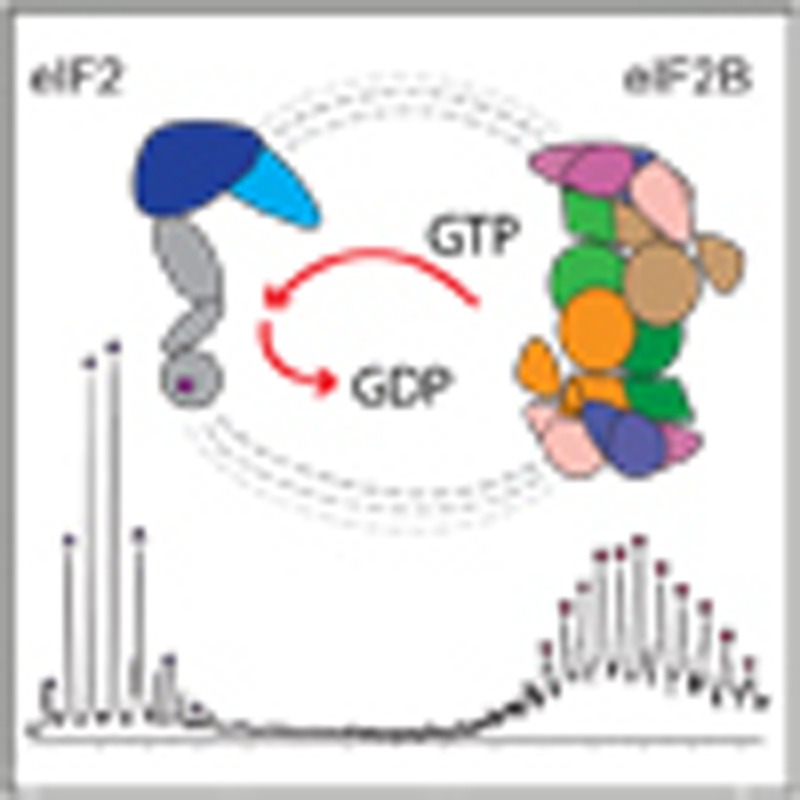

Protein synthesis in eukaryotes is a highly regulated process, particularly at the initiation phase, which requires the coordinated interplay of at least ten initiation factors (eIFs). Translation initiation begins with GTP-bound translation initiation factor 2 (eIF2) forming a ternary complex with methionyl initiator tRNA (Met-tRNA_i_) and delivering it to the P-site on the small 40S ribosomal subunit^1^. In association with other eIFs, this pre-initiation complex binds mRNA 5′-untranslated regions near the mRNA cap and scans downstream until it encounters an initiation AUG codon. Hydrolysis of eIF2-bound GTP and Pi release is triggered by correct basepairing of the Met-tRNA_i_ anticodon with the AUG codon of mRNA. This results in the dissociation of eIF2-GDP and other associated eIFs to permit 60S joining and transition to the elongation phase^2^. eIF2-GDP is released from the ribosome in complex with eIF5 (ref. 3), preventing the release of eIF2-bound GDP^4^ until eIF2B displaces eIF5 (ref. 5). eIF2B then acts as a guanine nucleotide exchange factor (GEF) catalyzing exchange of GDP for GTP, reactivating eIF2 and allowing subsequent rounds of translation initiation.

eIF2 is a well-conserved heterotrimer comprising α-, β- and γ-subunits, encoded by EIFS1-S3 in humans and *SUI2*, *SUI3* and *GCD11* in yeast. eIF2γ contains the GTP-binding site and together with β- and α-subunits binds Met-tRNA_i_^6^,^7^, whereas eIF2α is a major point of protein synthesis control. Alterations in protein synthesis, in response to diverse stresses such as viral infection or starvation, are mediated by phosphorylation of the α-subunit at Ser-51 (or Ser52 according to *SUI2* gene sequence; however, historically phosphorylation of eIF2α is referred to as Ser51, not taking into account the first methionine, which is post-translationally removed) by eIF2α kinases^8^. Phosphorylation converts eIF2 from a substrate for its GEF eIF2B to a competitive inhibitor^9^,^10^ and reduces the rate of translation initiation. It also activates translation of stress-response genes and is called the integrated stress response, as it provides a common node for multiple stresses. Diminished eIF2 phosphorylation response causes diseases in mammals ranging from metabolic disorders, to altered sensitivity to viral infection or altered brain functions, including enhanced long-term memory^1^. Clinical interest in these proteins has been raised because missense mutations within eIF2 and eIF2B subunits have been linked to distinct inherited neurodegenerative disorders^11^,^12^.

Detailed structural information for eIF2 is provided by X-ray crystallography of the archaeal homologue (aIF2) from *Sulfolobus solfataricus*^13^. In this structure, there is no contact between α- and β-subunits, which both bind the γ-subunit independently^13^. Less is known however about eukaryotic eIF2, the α-subunit being the best characterized^14^,^15^,^16^. An NMR solution structure of human eIF2α revealed three domains^14^, while the highly conserved loop containing Ser51 (the phosphorylation site) is well-resolved in the X-ray structure of the amino terminal region (2–175, domains 1 and 2) of yeast eIF2α^16^. In contrast to eIF2, the only atomic resolution structures of eIF2B subunits are of human eIF2Bα^17^, comprising an N-terminal α-helical domain (NαH) and the carboxy terminal domain with a Rossmann-like fold (RLF) and the C-terminal catalytic domain of the ε-subunit (ε-cat)^18^. No high-resolution structures are available for the remaining eIF2B subunits, for interactions between eIF2B subunits or between eIF2B and its substrate eIF2.

Considerable insight into the structure and function of eIF2B has been provided by genetic and biochemical methods, mostly applied to the yeast factor. Studies on the molecular basis for control of eIF2B by eIF2 phosphorylation have shown that three of the five eIF2B subunits (α, β and δ) comprise a regulatory subcomplex that can sense and respond to this important regulatory event by binding to eIF2α^9^. This binding is enhanced by eIF2α phosphorylation^19^ and abrogates the catalytic GEF activity of eIF2B. Single mutations in these subunits overcome regulation of eIF2B by eIF2 phosphorylation^20^, suggesting a broad interface between eIF2αP and eIF2B. Genetic co-depletion of β/Gcd7 and δ/Gcd2 allowed the formation of a subcomplex containing the remaining three subunits but weakened its interaction with eIF2 *in vivo*^21^. GEF function is accomplished by the γ- and ε-subunits (encoded by *GCD1* and *GCD6* in yeast) that form a subcomplex when co-overexpressed^19^,^22^. As indicated above, the C terminus of eIF2Bε bears the catalytic domain^23^ but it remains unclear how the γ-subunit contributes to GEF activity. Direct interactions between ε/GCD6 and eIF2β^24^, and ε/GCD6 and eIF2γ were determined by mutagenesis and pull-down assays^25^,^26^. Valuable insight into the interactions between catalytic eIF2Bε and γ-subunits has recently been reported by combining homology modelling, protein deletions and mutations. Combination of these techniques allowed identification of domain boundaries in catalytic subunits and their individual contributions to eIF2B complex formation in yeast^27^ and humans^28^. In yeast^27^, the catalytic subunits of eIF2B (γ and ε) share sequence similarity and domain structure with each other and with a family of phospho-hexose sugar-nucleotide pyrophosphorylases for which X-ray structures are available (ADP-glucose pyrophosphorylase (AGP) tetramer—(PDB 1YP2) and GlmU trimer (PDB 2OI7)). These structures exhibit different oligomeric states and relative arrangements of the pyrophosphorylase-like (PL) and left-handed β-helix (LβH) domains. In yeast, PL and LβH domains were shown to contribute independently to the interactions between γ and ε of eIF2B, favouring half of the AGP-tetramer arrangement over GlmU-like interactions^27^.

The dynamic nature of the eIF2:eIF2B interactions have so far resisted X-ray crystallography and require alternative methods. Mass spectrometry (MS) allows the study of protein complexes from solutions in which the native state is preserved, proving informative for determining subunit architectures and protein interactions^29^,^30^. Here we apply nano-electrospray MS and proteomics, coupled with chemical cross-linking, surface accessibility measurements and homology modelling to define subunit interactions within eIF2B, eIF2, as well as eIF2B and eIF2. We show that eIF2B exists as a decamer with its catalytic core γ_2_ε_2_ responsible for GTP binding and exchange. Our findings prompt us to propose a model for the subunit arrangement within the eIF2B decamer and provide new insights into its function as a GEF for eIF2.

## Results

### MS reveals an unexpected stoichiometry of eIF2B subunits

**Figure 1:**
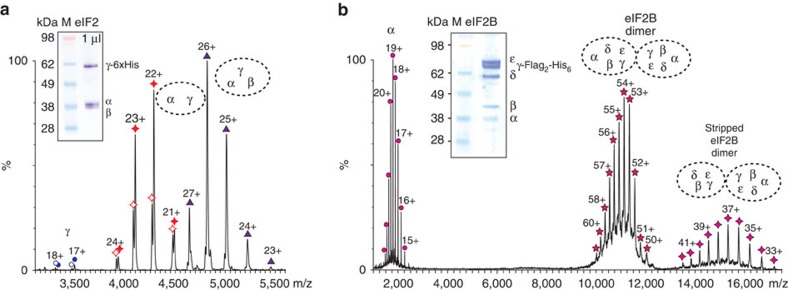
Native MS of yeast eIF2 and eIF2B complexes. (**a**) SDS–PAGE (insert) and mass spectrum of the purified yeast His-tagged eIF2 complex showing charge state distributions (labelled with ‘number of charges’ +) for the main species at 4,500–5,500 *m*/*z* corresponding to α/β/γ trimer (purple triangles). The α/γ dimer (red diamonds) at 4,000–4,500 *m*/*z* and γ at 3,200–3,500 *m*/*z* (blue circles) have peak splitting corresponding to a GDP molecule attached. (**b**) SDS–PAGE (insert) and mass spectrum of the purified yeast FLAG-tagged eIF2B complex showing the main species at 10,000–12,000 *m*/*z* corresponding in mass to the double of the eIF2B pentamer (pink star). High-collision energy results in dissociation of the α-subunit (pink circles) at 2,000 *m*/*z* and formation of the stripped complexes (14,000–16,000 *m*/*z*) where one α-subunit was lost from the eIF2B decamer (pink diamonds). The spectra shown represent an experiment from at least three biological replicates.

Mass spectra of eIF2 purified from a yeast strain co-overexpressing all three subunits shows that the dominant species has a molecular mass of 125 kDa, corresponding closely to the calculated mass of the α/β/γ eIF2 trimer (Fig. 1a). Peak splitting, resulting from a mass difference of 450 Da, is assigned to GDP binding (443 Da), present during purification and binding to the γ-subunit (Fig. 1a and Supplementary Table 1). The α/γ dimer (93.8 and 94.2 kDa) and the His-tagged γ-subunit (58.9 and 59.3 kDa), both with and without GDP, were also observed at low intensity.

The mass spectrum of FLAG-tagged eIF2B (similarly obtained by purification from a yeast strain engineered to co-overexpress all five subunits) shows a species of higher mass than expected for the eIF2B pentamer (298.0 kDa) (Fig. 1b). The measured mass (~\n600 kDa) corresponds to twice the anticipated mass of an eIF2B pentamer (596.0 kDa). No charge states were observed for a pentameric form, indicating that eIF2B exists exclusively as a decamer. Under high-collision energy conditions, one or two α-subunits were expelled from the eIF2B decamer (Fig. 1b and Supplementary Fig. 1). eIF2Bα is the only non-essential subunit of eIF2B^22^ and its loss during purifications of native protein complexes, requiring sequential column chromatography steps, has been observed previously^31^. Taken together with previous data, the MS analysis is consistent with the idea that α-subunits are located on the periphery of the eIF2B complex rather than within its core.

### Complex assembly and GTP binding involve eIF2Bγ and ε

**Figure 2:**
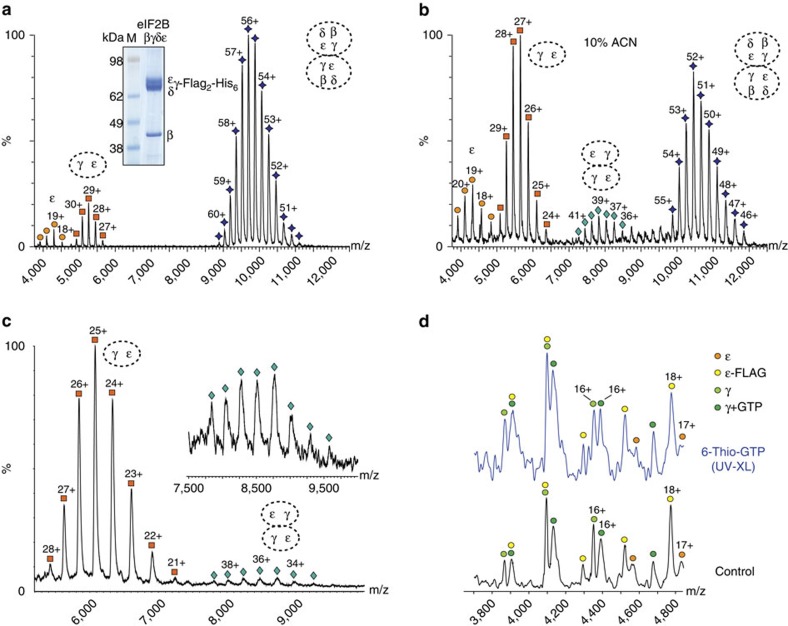
MS of eIF2B subcomplexes. (**a**) SDS–PAGE (insert) and mass spectrum of the purified yeast four subunit complex lacking the α-subunit eIF2Bβδγε, showing the main species at 9,000–11,000 *m*/*z*, corresponding in mass to octameric eIF2Bβδγε (purple diamonds); species at lower *m*/*z* correspond to the ε-subunit alone (orange circles at ~\n4,000 *m/*z) and γε dimer (orange squares at ~\n5,300 *m*/*z*). (**b**) Mass spectrum of the same eight subunit eIF2Bβδγε complex (purple diamonds) as in **a** after adding 10% ACN, showing appearance of an additional species at 8,000 *m*/*z* corresponding in mass to the γ_2_ε_2_ tetramer (green diamonds), suggesting its hydrophobic nature. Peaks for the γε dimer (orange squares) and ε-subunit (orange circles) are also increased after disruption with 10% ACN. (**c**) Mass spectrum of the γε core complex of eIF2B showing charge state distributions for the γε dimer (~\n6,000 *m*/*z*, orange squares) and the γ_2_ε_2_ tetramer (~\n8,500 *m*/*z*, green diamonds). (**d**) Mass spectrum of the low *m*/*z* region of the same spectrum as in **c** (lower panel) and after incubation with 6-Thio-GTP and ultraviolet cross-linking (upper panel). Separately purified ε-subunit (FLAG-tagged, yellow circles) has been added as a control. Intensities of GTP-bound eIF2Bγ (dark green circles) increase after incubation with 6-Thio-GTP and ultraviolet cross-linking. The spectra shown represent an experiment from two biological replicates.

To address the question of which eIF2B subunits are involved in complex assembly, we purified FLAG-tagged eIF2B lacking the α-subunits. Mass spectra showed that this complex is an octamer with a mass of 532.2 kDa (Fig. 2a). Tandem MS (MS/MS; 57+ charge state) of the complex showed that β-subunits dissociate readily (Supplementary Fig. 2), indicating their probable peripheral location. In-solution disruption experiments were carried out with organic solvents to disrupt hydrophobic interfaces^29^,^32^. Addition of 10% acetonitrile (ACN) to the complex-containing solution resulted in the formation of a tetramer of γ- and ε-subunits (305.2 kDa) (Fig. 2b), suggesting that γ- and ε-subunits form a tetrameric core of the complex, with β- and δ-subunits released from the octamer following disruption of hydrophobic interfaces.

To confirm the proposed tetrameric core, we purified the eIF2Bγε complex applying the same FLAG-tag strategy (using FLAG-tagged γ-subunit)^33^. We found that the γε-core is present as dimeric and tetrameric complexes (Fig. 2c), with the dimeric species being predominant. This implies that β- and δ-subunits stabilize the γ_2_ε_2_-core in the intact eIF2B complex. Interestingly, the masses of both complexes (γε and γ_2_ε_2_) were significantly higher than predicted by summing the masses recorded for ε- and γ-subunits present at lower *m*/*z* (Fig. 2d). The mass differences between theoretical and observed masses (832 and 2,830 Da) suggest binding of one or more metabolites to either the ε- or γ-subunits.

Prior analysis has indicated that GTP can bind to eIF2B^34^, and as pyrophosphorylase enzymes including AGP bind specific sugars and nucleotides^27^, we hypothesized that either the ε- or γ-subunit PL domains might bind GTP. As GTP binding by eIF2B could contribute to its nucleotide exchange activity, we therefore investigated GTP binding to the γ_2_ε_2_-core complex or to FLAG-tag purified ε-subunit alone by addition of 6-Thio-GTP, a photo-reactive base analogue of GTP. The low *m*/*z* region of the spectrum shows the ε-subunit, FLAG-tagged ε- (purified separately) and γ-subunits. Importantly, two populations of γ-subunit were observed, one with an additional mass of 637 Da, corresponding to binding of one GTP. After incubation with 6-Thio-GTP and ultraviolet irradiation, the intensity of the GTP-bound population of the γ-subunit increased, while for both ε-subunits (with and without FLAG-tag) no nucleotide binding could be observed (Fig. 2d). These data provide strong evidence that eIF2Bγ, but not eIF2Bε, can bind GTP.

### Transient interactions between eIF2 and eIF2B

We applied two different strategies to form eIF2–eIF2B complexes and transfer them intact to the gas phase. First, we used a purification procedure of eIF2B with a FLAG-tagged γ-subunit^33^ under low ionic strength conditions to retain interactions with eIF2. All subunits were observed on SDS–polyacrylamide gel electrophoresis (SDS–PAGE) gels (Supplementary Fig. 3a, insert) with eIF2 subunits being sub-stoichiometric to eIF2B subunits as judged by the intensity of stained gel bands. The identity of all proteins was confirmed by nanoflow liquid chromatography-coupled tandem mass spectrometry (nano LC-MS/MS) of tryptic peptides. Mass spectra of the eIF2–eIF2B complex purified at low ionic strength showed charge states for the eIF2B decamer (11,500–13,000 *m*/*z*) and the eIF2 trimer (5,000–6,000 *m*/*z*) (Supplementary Fig. 3a). Again, no charge states were observed for a pentameric form of eIF2B. Other species corresponding to the eIF2 α/γ dimer, eIF2γ, eIF2Bγ and eIF2Bγε were also present in the spectra. The eIF2–eIF2B complex was retained during purification but the absence of an intact eIF2:eIF2B complex in mass spectra confirms that interactions are transient.

Attempting to stabilize these transient interactions in eIF2:eIF2B, we purified γ-His tagged eIF2 phosphorylated *in vitro* by human PKR, as described previously^35^. Phosphorylation of eIF2α-Ser51 was confirmed by LC-MS/MS of tryptic peptides. Increasing ratios of phosphorylated eIF2 were then added to eIF2B immobilized on anti-FLAG resin and the complexes eluted with a FLAG-peptide. This procedure resulted in a greater proportion of eIF2 purifying with eIF2B, as judged by the intensity of gel bands (Supplementary Fig. 3b insert), and is consistent with enhanced binding of eIF2 to eIF2B induced by eIF2α phosphorylation. Mass spectra of this phosphorylated eIF2–eIF2B complex however revealed the eIF2 and the eIF2B decamer as separate species, very similar to the low ionic strength preparation (Supplementary Fig. 3). The fact that we are able to purify an eIF2–eIF2B complex, via a FLAG-tagged γ-subunit of eIF2B, and to enhance eIF2 binding after eIF2α phosphorylation confirms their interaction during purification. The fact that the complex also readily dissociates before MS, suggests that the interactions between these two protein complexes are of a transient nature.

### Subunit stoichiometry of eIF2–eIF2B complexes

As we have established individual eIF2 and eIF2B stoichiometries but could not detect the intact eIF2–eIF2B complex we used label-free quantitative proteomics to determine absolute quantities of protein subunits and thus stoichiometries of interacting eIF2 and eIF2B proteins. The eIF2–eIF2B complex was purified after phosphorylation of eIF2 as described above and proteins were digested with trypsin and subjected to nanoLC-MS/MS. Abundances of the various subunits in the complex were determined by intensity-based absolute quantification (iBAQ)^36^, which takes into account the sum of peak intensities of all the peptides ascribed to a specific protein divided by the number of theoretical peptides. Results of the quantification (Supplementary Fig. 4) show that α- and β-subunits of eIF2 are close to stoichiometric with γ showing higher abundance likely to be due to the presence of His-tag used for eIF2 purification (α:β:γ corresponding to 1:1.08:1.67). For eIF2B subunits quantified by iBAQ, the abundance of α, ε and δ (1.12:0.76:0.95) correlates well with the FLAG-tagged subunit γ (set to 1), whereas the β-subunit ratio was slightly higher than anticipated (1.4-fold over γ). On average, eIF2B:eIF2 subunits were 4:1 in line with the proposal that eIF2 is sub-stoichiometric, due to its transient interactions with eIF2B.

### Chemical cross-linking defines eIF2–eIF2B interactions

Interactions between protein subunits within the eIF2 and eIF2B complexes, as well as in the eIF2:eIF2B complex, were assessed by chemical cross-linking using deuterated (d4) and non-deuterated (d0) (BS3) cross-linker (Methods). Different complexes were cross-linked: the eIF2:eIF2B complex, the four-subunit eIF2B complex (lacking the α-subunit), the eIF2Bγε core complex, and eIF2:eIF2Bε and eIF2:eIF2ε-cat subcomplexes. In total, we identified 167 unique cross-links (Supplementary Tables 2–5) within all (sub-) complexes. These included 36 inter subunit cross-links, of which 11 were assigned between eIF2B and eIF2.

**Figure 3:**
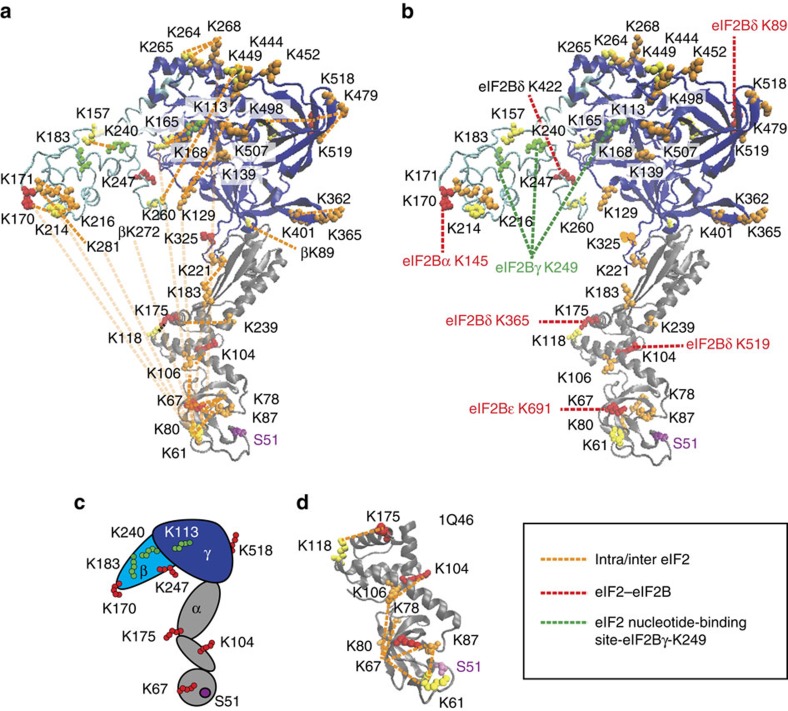
Homology model of yeast eIF2 with identified cross-links. (**a**) Homology models for eIF2 α- (grey), β- (cyan) and γ- (blue) subunits were obtained from Swiss Model modelling server and are based on the crystal structure of archaeal aIF2 from *S. solfataricus* (PDB 3CW2) used as a template. The highly dynamic and flexible nature of eIF2 is apparent from the multiple cross-links observed. Cross-linked lysine residues are shown as coloured spheres, and identified intra- and inter-protein cross-links within eIF2 are shown as orange dashed lines; eIF2α Ser51, involved in regulation, is shown in magenta. (**b**) Homology models as shown in **a**. Inter-protein cross-links between eIF2 and eIF2B (red), and inter-protein cross-links between eIF2Bγ K249 and residues around nucleotide-binding site of eIF2 (green). (**c**) Cartoon representation of eIF2 with mapped inter-cross-linked residues. (**d**) Crystal structure of the N-terminal part of the eIF2 α-subunit from *S. cerevisiae* (PDB 1Q46) with identified cross-links and containing Ser51 (labelled as in Fig. 3a). Cross-links shown were obtained from different cross-linking experiments (Supplementary Tables 4 and 5).

**Figure 4:**
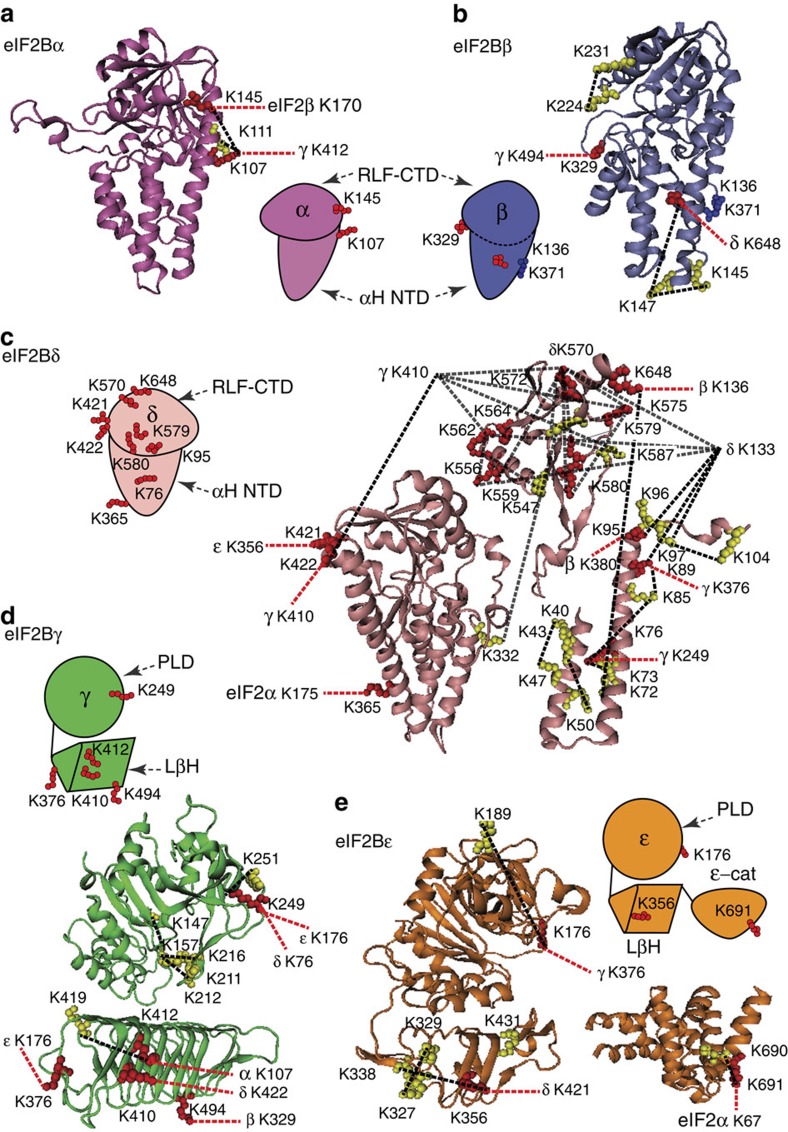
Homology model of yeast eIF2B subunits with identified cross-links. (**a**) Homology model of the eIF2Bα (res. 1–304) based on the crystal structure of human eIF2Bα (PDB 3ECSA) and cartoon representation of the α-subunit with mapped intra- (yellow) and inter- (red) cross-linked residues. Cross-links are represented by dashed lines: intra, black; inter, red. (**b**) Homology model of the eIF2Bβ (res. 60–371) based on the crystal structure of human eIF2Bα (PDB 3ECSA) and cartoon representation of the β-subunit with mapped cross-linked residues (labelled as in Fig. 4a). (**c**) Homology models of the eIF2Bδ: residues 1–244 based on PDB 1YA9A template; residues 245–536 based on PDB 2YVKA template; residues 540–651 based on PDB 3A11A template and cartoon representation of the δ-subunit with mapped cross-linked residues (labelled as in Fig. 4a). (**d**) Homology models of the eIF2Bγ PL domain (res. 44–314) and LβH (res. 358–578) domains based on PDB 1YP2D and 2OI7A templates, respectively, and cartoon representation the γ-subunit with mapped cross-linked residues (labelled as in Fig. 4a). (**e**) Homology model of the eIF2Bε (res.30–431) based on PDB 1YP2A; the crystal structure of the catalytic domain ε-cat (res. 524–712) from *S. cerevisiae* (PDB 1PAQ) and cartoon representation of the ε-subunit with mapped cross-linked residues (labelled as in Fig. 4a). Cross-links shown were obtained from different cross-linking experiments (Supplementary Table 2).

To place our cross-links in a structural context, we used homology models (Supplementary Table 6) generated by MODELLER^37^ and SWISS-MODEL^38^ web servers for yeast eIF2 and eIF2B subunits (Figs 3a and 4a–e). Using intra-protein cross-links (Supplementary Tables 2 and 4) assigned to individual subunits, we validated our cross-linking strategy, finding a good agreement with the folds of the homologous structures generated and the distances between intra-cross-linked residues measured^39^ (Figs 3a and 4a–e).

The available crystal structure of the yeast eIF2α subunit is incomplete (PDB 1Q46, Fig. 3d)^16^ and we therefore generated a new homology model for this subunit. Both eIF2 α- and β-subunits were modelled using the crystal structure of archaeal aIF2 from *S. solfataricus* (PDB 3CW2) as a template. eIF2γ was modelled using the crystal structure of eIF2γ from *Pyrococcus abyssi* (PDB 1KK1A). Homology models for all three yeast eIF2 subunits were then aligned with the crystal structure of *S. solfataricus* (PDB 3CW2) (Fig. 3a). Modelled structures of α- (residues 7–267) and γ- (residues 96–527) subunits were almost complete, while the model of the β-subunit is missing the amino-terminal domain (NTD) residues 1–126 in which three cross-links with the γ-subunit were found (βK19 or βK86 to γK184 and βK89 to γK401; Supplementary Table 4). Cross-linked peptides identified within eIF2 are in good agreement with both the crystal structure of the yeast α-subunit (Fig. 3c) and the fold of the homologous structures (Fig. 3a). One exception is the βK260-γK449 cross-link and implies that the C-terminal end of the β-subunit (βK260) is flexible enough to come into the vicinity of γK449. Another exception is domain I of eIF2α, where K61 cross-links multiple times with the C-terminal half of the β-subunit (Supplementary Table 4) and the γ-subunit G domain (Fig. 3a). The first two domains of eIF2α and the C-terminal region of eIF2β are thought to be highly mobile^40^, making many transient dynamic interactions, which probably explains these crosslinks.

### eIF2B subunit domain arrangements based on inter-cross-links

Similar to eIF2, we generated homology models for eIF2B subunits (Supplementary Table 6). The majority of these, however, are based on homologous structures of proteins with different or unknown functions and lower sequence identity than was observed for eIF2. For some proteins, we could only generate homology models for separate domains rather then full-length proteins. We therefore mapped intra-subunit cross-links at a domain level (Fig. 4a–e).

**Figure 5:**
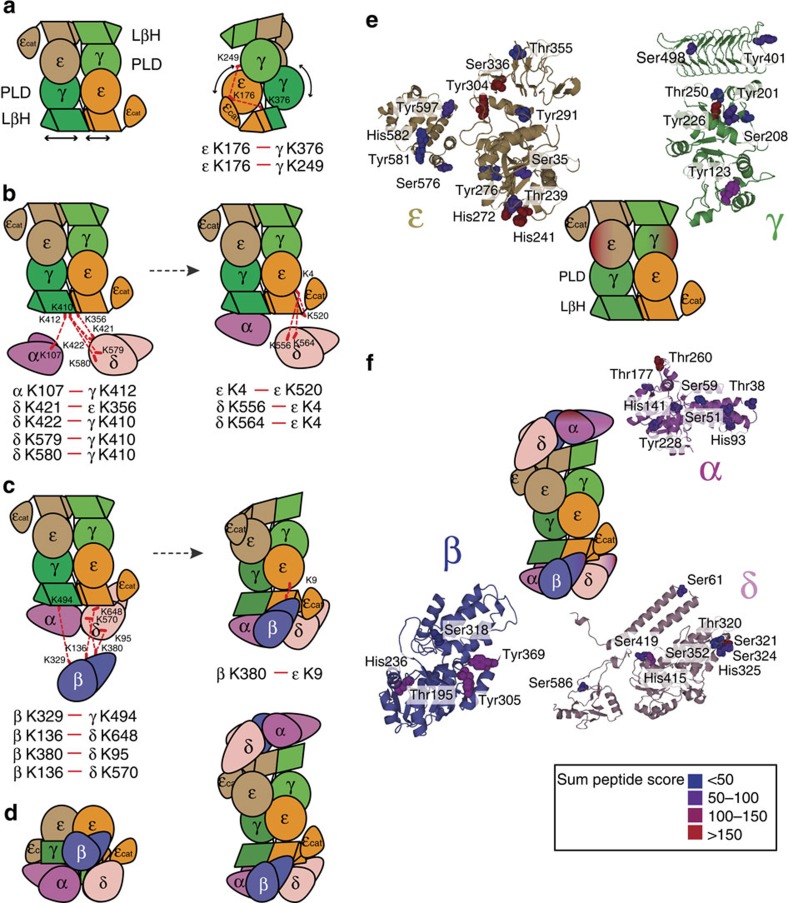
Assembly of eIF2B subunits based on identified cross-links and solvent accessibility. (**a**) Schematic representation of the γ_2_ε_2_ hydrophobic core showing PL and LβH subunit domain arrangement resembling the arrangement of the AGP homo-tetramer: two copies of γ-subunit are shown in light and dark green, and two copies of ε in orange and beige. LβH domain in the γ-subunit is shown slightly longer than in the ε-subunit (left). Right: cartoon representation of the rotated view of the γ_2_ε_2_ hydrophobic core with identified cross-links (red dashed line) between εK176 and γK376, and γK249 (right). (**b**) Left and right: cartoon representation of the γ_2_ε_2_ hydrophobic core and α- and δ-subunits with cross-links (red dashed lines) to the LβH domains of γ and ε. (**c**) Left and right: cartoon representation of the γ/ε hydrophobic core, α- and δ-subunits and the cross-links of the β-subunit (red dashed lines) to the LβH domains of γ and ε and RLF domain of the δ-subunit. (**d**) Schematic model of eIF2B decamer shows the tetrameric γ_2_ε_2_ core with a regulatory subcomplex of α/β/δ trimers attached to the core through the interaction with ε and γ LβH domains shifted towards ε (right). This shift facilitates interactions of β and δ with the ε PL domain. View from the bottom of the regulatory sub complex α/β/δ is shown (left). (**e**) Homology models for eIF2Bε and γ subunits are shown and labelled residues are represented as space fillings. The sum of the peptide score obtained for labelled peptides is given in different colours and labelled sites are shown accordingly. Solvent accessibility of protein subunits is indicated in the cartoon representation of eIF2B. (**f**) Homology models for eIF2Bα, β and δ subunits are shown and labelled residues are represented as in **e**. Cross-links shown were obtained from different cross-linking experiments (Supplementary Tables 2 and 3).

Based on our findings that γ- and ε-subunits probably form a tetrameric hydrophobic core, we propose that γ- and ε-subunits interact to form a γ_2_ε_2_ hetero-tetramer, resembling the arrangement of the structural homologue—homotetramer ADP-glucose pyrophosphorylase (AGP). Similar to AGP, these eIF2B subunits both have an N-terminal PL domain and a second domain that is predicted to adopt an LβH (Fig. 4d,e). This arrangement agrees well with those proposed in recent studies mapping domain interactions between these two subunits by complementary methods^27^,^28^ and with identified cross-links between γK249 and εK176 (Fig. 5a). ε-cat is shown adjacent to the εLβH as it is not required for interactions with other eIF2B subunits^23^,^27^.

Two cross-links between adjacent residues in the eIF2Bδ RLF domain (K421 and K422) to 2BεK356 (LβH) and 2BγK410 (LβH), respectively, indicate that residues εK356 and γK410 are proximal, as expected when γ and ε interact via their LβH domains, and locate eIF2Bδ relative to the γ_2_ε_2_ core (Fig. 5b). Position of the *δ*-subunit is further constrained by residues K579 and K580 in its RLF domain, proximal to K422 in the homologous structure, cross-linked to γK410. Two further cross-links in the δ carboxy-terminal domain (CTD) RLF domain δK556 and δK564 to εK4, which in turn is cross-linked to the εK520 at the end of ε-LβH, suggest that the δ-subunit is inclined towards the ε-subunit (Fig. 5b).

Arrangement of eIF2Bα is constrained by the cross-link between αK107 (NαH) and γK412 (LβH), two residues apart from γK410, which cross-links to δK422, restricting the position of eIF2Bα, not only relative to γ- but also to the δ-subunit (Fig. 5b). The cross-linked residues βK380 and εK9 of eIF2B are not present in the homology models; however, approximate location of the eIF2Bβ can be inferred based on available residues in close proximity (βQ371 and εK4, Fig. 4b). The location of the β-subunit is further confirmed by cross-links K136 and K380 to the δ-subunit residues K648 and K95, and the cross-link between βK329 and γK494 (Fig. 5c). These constraints position the β-subunit relative to α- and δ-subunits, which we propose form a trimer by extensive interactions between their RLF domains in agreement with previous studies, which demonstrate interactions between α, β and δ to form an eIF2 phosphorylation-sensing regulatory subcomplex^9^,^41^ (Fig. 5d).

We completed our structural model of the eIF2B decamer by symmetry, reflecting the organization of subunits on a protein domain level (Fig. 5d). We assembled the regulatory subunits α, β and δ to form asymmetric trimers interacting through RLF domains with NαH domains oriented outwards (Fig. 5c). Binding of the regulatory subcomplex to the γ_2_ε_2_ core occurs through both ε and γ LβH domains. β- and δ-subunits are inclined towards the ε-subunit, providing more extensive interactions with ε LβH and extending to form interactions with the ε PL domain.

### Solvent accessibility measurements

To provide complementary evidence for the eIF2B subunit arrangement, we probed the exposed surfaces of intact eIF2B by chemically labelling. We subjected the eIF2B complex to the labelling reagent (diethylpyrocarbonate (DEPC)) for 1 min. Following enzymatic digestion and assignment of the labelled peptides using nano LC-MS/MS, we located the solvent accessible residues modified by the labelling reagent, onto the proposed subunit structure models (Fig. 5e,f). Within the catalytic domain, for which a structure is available, the surface residues identified reassuringly map close to the identified catalytic residue E569 (ref. 18), showing this region is accessible for eIF2 interaction. The results also confirm that our proposed interaction interfaces between γ- and ε-subunits are not modified by the labelling reagent and so support our proposed model of the γ_2_ε_2_ core complex (Fig. 5e). The crystal structure for eIF2Bα revealed a strong dimer interface along one hydrophobic edge of the RLF domain (pdb 3ECS). The chemical labelling approach showed that although this subunit is generally solvent accessible, this hydrophobic RLF edge is not, suggesting a general peripheral location for eIF2Bα within the complex, but allowing interaction via the RLF hydrophobic edge. Similarly, the eIF2Bβ and *δ*-subunit solvent accessibility measurements support a model placing them at the exterior of the protein complex (Fig. 5f).

### Model for eIF2–eIF2B interactions based on cross-links

**Figure 6:**
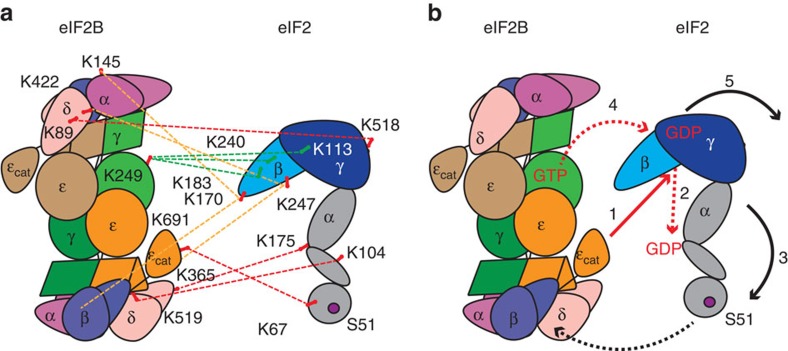
Interactions between eIF2 and eIF2B identified by cross-links. (**a**) Schematic representation of the eIF2 and eIF2B modelled structure with mapped inter-subunit cross-links (red dashed lines); inter-cross-links between residues βK183, βK240 and γ K113 of eIF2, in close proximity to the nucleotide binding pocket, to the eIF2Bγ K249 located in the vicinity of the potential nucleotide binding site (green dashed lines); inter-cross-links of eIF2β (K170 and K247) to either of the two eIF2Bα (K145) and eIF2Bδ (K422) subunits (orange dashed lines). Cross-links shown were obtained from different cross-linking experiments (Supplementary Table 5). (**b**) Schematic model of eIF2 and eIF2B interactions based on homology modelling and identified cross-links, proposing that GEF function of eIF2B is a multi-step process whereby ε-cat of eIF2B promotes GDP release from eIF2γ (1 and 2) possibly followed by a conformational change (3) allowing transfer of the GTP residing in the nucleotide pocket of eIF2Bγ PL domain (4) and subsequent dissociation of eIF2 (5) after another conformational change induced by GTP binding to eIF2γ. Tighter binding of eIF2 to eIF2B on eIF2α phosphorylation is very likely to interfere with the conformational changes necessary for catalyses abrogating eIF2B function.

As eIF2B is a dimer with two catalytic domains, it has the potential to interact with two molecules of eIF2. Similarly, a single eIF2 trimer has the potential to interact with residues from both copies of each eIF2B subunit. Therefore, our proposed ‘working model’ represents one possible solution to this complex problem derived from the cross-linking data we obtained, rather than a definitive interaction model. To position eIF2 relative to eIF2B in the eIF2:eIF2B complex we used five critical cross-links: eIF2α K175:eIF2Bδ K365 and eIF2α K104:eIF2Bδ K519 (red dashed lines, Fig. 6a) together with eIF2β K183, β K240 and γ K113 to eIF2Bγ K249 (green dashed lines, Fig. 6a). The cross-links between eIF2α and eIF2Bδ subunits bring the N-terminal domain of eIF2α containing Ser51 close to the interface between β- and δ-subunits of eIF2B. Although the other three eIF2 residues are close to the GDP/GTP nucleotide-binding site of the γ-subunit of eIF2, eIF2Bγ K249 is located in the PL domain and the observed cross-links place it in close proximity to the nucleotide-binding pocket of eIF2γ.

Other cross-links between eIF2 and eIF2B confirm this arrangement. For example, eIF2γ K518 cross-links to K89 of eIF2Bδ located on the same side as eIF2Bγ, involved in interactions with eIF2γ. Taking into account that eIF2β is flexible and that it is not possible to identify the structural arrangement of subunits at the atomic level, we cannot determine which one of the two eIF2Bα K145 residues are cross-linked to eIF2β K170 (yellow dashed lines, Fig. 6a). Similarly, the cross-link between eIF2β K247 and eIF2Bδ K422 could be formed to either of the two eIF2Bδ subunits. In agreement with our proposed model, we have identified cross-links between the catalytic domain of eIF2Bε (K691) and eIF2α (K67) (Fig. 6a). We have further cross-linked eIF2–eIF2Bε subcomplex comprising (i) full-length ε-subunit and (ii) the catalytic domain of ε (Supplementary Fig. 5). Cross-links observed in these complexes confirm interactions between the catalytic ε-subunit and eIF2. Additional intra-cross-links (Supplementary Table 4) in eIF2 subunits that are not present when eIF2 is in complex with decameric eIF2B suggest that these residues may be shielded by the interaction with eIF2B subunits (Fig. 3). A similar explanation may account for our inability to identify any cross-links between the catalytic domain of ε-subunit and eIF2γ.

## Discussion

In this study we assessed the composition of eIF2 and eIF2B complexes and their interactions by MS coupled with chemical cross-linking and quantitative proteomics. Although it is generally accepted that eIF2B is composed of five non-identical subunits in unit stoichiometry in yeast^19^ and human^28^, we show that this complex exists as a decamer in 2:2:2:2:2 stoichiometry in line with very recent results for human eIF2B^42^. We used a BS3-d_0_/d_4_ cross-linker to determine interaction sites between eIF2B subunits, as well as interactions between eIF2B and eIF2. Based on these results, together with homology modelling, we present a model of subunit interactions within the eIF2B decamer and also its interactions with eIF2. We propose that the assembly of the eIF2B decamer occurs through the formation of a γ_2_ε_2_ tetrameric core, resembling the subunit arrangement in AGP homo-tetramer. The *α*-, *β*- and *δ*-subunits are arranged in asymmetric trimers associated to the core through ε and γ LβH domains. This arrangement is in agreement with prior mutagenesis and pull-down studies demonstrating that both ε and γ LβH domains mediate the interactions between the catalytic and regulatory subcomplexes in both yeast and human eIF2B^27^,^28^.

We found that the catalytic γ_2_ε_2_-core has an additional mass bound, that may include GTP and showed directly that GTP can bind to the γ-subunit, but not to the ε-subunit. As pyrophosphorylase enyzymes bind specific nucleotides (for example, AGP binds ATP), it is reasonable to propose that the eIF2Bγ PL domain within its N-terminal half binds GTP. This idea is supported by site-directed mutagenesis around the nucleotide-binding site in yeast ε PL domain, which has only modest effect on eIF2B activity^27^. By contrast, mutation of Tyr-38, close to the putative nucleotide-binding site in human eIF2Bγ, severely impairs catalytic function^28^, implying possibly that GTP binding to eIF2Bγ is important for its function. Two models for GTP binding to eIF2B have been proposed previously. First, an allosteric role has been suggested in which GTP binding is not directly involved in the catalytic mechanism, but instead acts as a signal that energy in the form of GTP is available to support continued protein synthesis. The second idea is that GTP binding to eIF2Bγ acts as a direct source of GTP for nucleotide exchange. This latter idea is supported by prior enzyme kinetic analysis with the yeast factor^43^ and by our cross-linking result that eIF2Bγ K249 (within the PL domain) crosslinks to eIF2γ K113 (within the G domain), thereby placing the GTP-binding sites for each factor in close proximity to each other (Fig. 6a). We favour this latter idea.

Based on our findings and taking into account other data, we propose that eIF2B assembly provides not only the catalytic function for guanine nucleotide exchange but also acts as a scaffold to hold eIF2 in place during catalysis. The eIF2B decamer of ~\n600 kDa may be capable of interacting with and catalysing exchange on two eIF2 complexes, simultaneously, but for simplicity we show only a single eIF2 here. From our model of eIF2–eIF2B interactions (Fig. 6a) and our identification of a GTP-binding site on eIF2Bγ, it seems likely that guanine nucleotide exchange involves several steps and these ideas are shown schematically in Fig. 6b. As proposed previously, initial binding of eIF2-GDP to eIF2B allows the catalytic domain of ε to reach the interface between β- and γ-subunits of eIF2 (Fig. 6b-1) and to promote GDP release from eIF2γ (Fig. 6b-2)^25^,^26^. By analogy with other G proteins, GDP release probably involves conformational changes in eIF2 subunits (Fig. 6b-3) followed by subsequent binding of GTP (Fig. 6b-4). The novel aspect of our model is that GTP is transferred from eIF2Bγ to the vacant site on eIF2γ (Fig. 6b-4). As the affinity of eIF2 for GDP is much higher than for GTP^43^ an immediate transfer of GTP to the vacated site on eIF2γ might be necessary. It seems reasonable to speculate that GTP binding to eIF2γ may then induce further conformational changes and dissociation of the two factors (Fig. 6b-5). Such a mechanism fits with the ‘substituted’ rather than ‘sequential’ mechanism for guanine nucleotide exchange, which is supported by prior enzyme kinetic analyses^34^. A sequential enzyme mechanism assumes that GDP is released from the eIF2-GDP:GEF complex before binding of GTP and GEF dissociation, whereas the substituted mechanism proceeds by the formation of a ternary complex containing both substrates eIF2:GDP:GTP:GEF before release of any the products^34^,^44^.

If we consider eIF2B being a platform for eIF2, accommodating conformational changes during catalysis, this may explain the regulatory mechanism of eIF2α phosphorylation at residue Ser51. Tight binding of the phosphorylated Ser51 to regulatory subunits is likely to interfere with conformational changes in eIF2 necessary for catalysis and, therefore, would abrogate eIF2B function. According to our model, Ser51 binds at the interface between β- and δ-subunits of eIF2B, in line with extensive prior genetic and biochemical studies^19^,^20^,^25^,^41^,^45^. In our model, eIF2Bα stabilizes interactions within the regulatory sub-complex making the structure more rigid. Therefore, mutations within the eIF2Bα subunit, or its elimination from the complex, may increase spatial flexibility of β and δ to allow conformational changes in eIF2 and permit nucleotide exchange.

Overall, our study advances understanding of the structural arrangement of the eIF2B complex and defines its interactions with eIF2. We have shown that eIF2B exists as a decamer formed around a tetrametic catalytic core. This hydrophobic γ_2_ε_2_ core suggests that in an evolutionary context the catalytic function may have preceded its regulatory role. The regulatory function, enabled by the later addition of the regulatory subunits resulting in the structural intricacy described here, making eIF2B one of the most complex GEFs known.

## Methods

### Protein expression and purification

Yeast *Saccharomyces cerevisiae* strains engineered to overexpress eIF2, eIF2B, eIF2Bε, eIF2Bε-cat and four-subunit eIF2B complex (lacking α-subunit) employed in this study were—GP3511 (*MAT*α *leu2-3 leu2-112 ura3-52::HIS4-lacZ ino1 gcn2*Δ *pep4::LEU2 sui2*Δ pAV1089[*SUI2 SUI3 GCD11*-His6 2 μm *URA3*])^19^, GP4109 (*MAT*α *leu2-3 leu2-112 ura3-52 ino1 gcd6*Δ *gcn2*Δ::hisG *ura3-52::HIS4-lacZ* pAV1428[*GCD6 GCD1*-FLAG2-His6 *URA3* 2 μm] pAV1494[*GCN3 GCD2 GCD7 LEU2* 2 μm])^23^, GP3915 (*MAT*α *trp1*Δ*63 ura3-52 leu2-3 leu2-112 GAL2+ gcn2*Δ *pep4::LEU2* pAV1427[FLAG-His6-*GCD6* 2 μm]), GP3977 (*MAT*α *trp1*Δ*63 ura3-52 leu2-3 leu2-112 pep4::LEU2 GAL2+ gcn2*Δ pAV1689[FLAG-His6-*GCD6*-518-712, *URA3*, 2 μm]) and GP4305 (*MAT*α *prb1-1122 pep4-3 leu2 trp1 ura3-52 gal2* pAV1413 [*GCD1*-FLAG2-His6 *GCD6* 2 μm *LEU2*] pAV1353[*GCN3 GCD2 GCD7* 2 μm *URA3*])^9^, respectively.

His-tagged yeast eIF2 was purified using Ni-chelate chromatography followed by chromatography on heparin-Sepharose^16^. FLAG-tagged eIF2B complexes and subunits were purified in a single affinity step in a high-salt buffer (500 mM KCl) according to ref. 33, with minor modifications for the five-subunit eIF2B: Briefly, after immobilizing the protein on anti-FLAG resin, we used 500 mM ammonium acetate as a wash and elution (containing FLAG peptide) buffer to reduce manipulation steps and time before native MS, which requires transfer of a protein complex to a volatile buffer (such as ammonium acetate) for efficient ionization in the electrospray source of a mass spectrometer. The eluted fraction (~\n400 μl) was concentrated in an Amicon Ultra (100 K MWCO) 0.5 ml centrifugal filter (Millipore) and washed ten times with 400 μl of 500 mM ammonium acetate to remove FLAG peptide, and then concentrated to a final volume of 50 μl. Three microlitres of the final sample were withdrawn for SDS–PAGE analysis.

### eIF2 phosphorylation and formation of eIF2–eIF2B complexes

Purified eIF2 was phosphorylated *in vitro* by human PKR (Invitrogen)^35^. eIF2-eIF2B complexes were formed by adding increasing amounts of phosphorylated eIF2 to eIF2B immobilized on anti-FLAG and and prewashed with phosphorylation buffer (20 mM Tris (pH 7.5), 100 mM KCl, 10 mM MgCl_2_, 5 mM β-glycerophosphate, 2 mM dithiothreitol, 10% glycerine, 0.1% NP-40, 200 μM ATP). The mixture was incubated constantly rotating at RT for 30 min. The eIF2-eIF2B complex was washed with 500 mM ammonium acetate and eluted with FLAG peptide. Eluted fractions were additionally washed with 500 mM ammonium acetate and concentrated in an Amicon Ultra (100 K MWCO). Low salt (100 mM KCl) purification of FLAG-tagged eIF2B resulted in maintaining interactions with eIF2 and eIF2B:eIF2 complex was eluted from anti-FLAG resin^19^.

### MS of intact eIF2 and eIF2B protein complexes

For intact MS protein containing solutions were exchanged into 500 mM ammonium acetate using Amicon centrifugal devices. MS and MS/MS spectra were acquired on a high mass quadrupole time-of-flight-type instrument^46^ adapted for a QSTAR XL platform (MDS Sciex)^47^ using in-house prepared gold-coated glass capillaries^26^. Optimized instrument parameters were as follows: ion spray voltage 1.3 kV, declustering potential 100 V, focusing potential 200 V and collision energy up to 200 V, MCP 2,350 V. In MS/MS, the relevant *m*/*z* range was selected in the second quadrupole and subjected to acceleration in the collision cell.

### Digestion with trypsin

The proteins were separated by gel electrophoresis using the NuPAGE gel system (Invitrogen) according to the manufacturer’s protocol. Protein bands of interest were cut and the proteins therein were digested in-gel as previously described^48^. Alternatively, the proteins were precipitated with ethanol and the protein pellet was dissolved in 10 μl 1% (m/v) RapiGest SF Surfactant (Waters). The proteins were reduced by addition of 10 μl 25 mM dithiothreitol and incubation at 37 °C for 1 h. Free cysteine residues were alkylated with 10 μl 100 mM iodoacetamide at 37 °C for 1 h. The RapiGest concentration was reduced to 0.1% (m/v) by addition of 25 mM ammonium bicarbonate. The proteins were digested with 0.5 μg Trypsin (Promega) at 37 °C overnight. RapiGest was decomposed by addition of 5% (v/v) trifluoroacetic acid and removed by centrifugation. The peptides were dried in a vacuum centrifuge.

### Nano LC-MS/MS

Tryptic peptides were separated by nano-flow reversed-phase LC using a DionexUltiMate 3,000 RSLC nano System (Thermo Scientific). Mobile phase A was 0.1% (v/v) formic acid (FA) and mobile phase B contained 80% (v/v) ACN/0.1% (v/v) FA. The peptides were loaded onto a trap column (2 cm, HPLC column Acclaim PepMap 100, C18, 100 μm I.D. particle size 5 μm; Thermo Scientific) and separated with a flow rate of 300 nl min^−1^ on an analytical C18 capillary column (50 cm, HPLC column Acclaim PepMap 100, C18, 75 μm I.D. particle size 3 μm; Thermo Scientific), with a gradient of 5–80% (v/v) mobile phase B over 74 min.

The nanoLC system was coupled to an LTQ-Orbitrap XL hybrid mass spectrometer (Thermo Scientific) and peptides were directly eluted into the mass spectrometer. Typical mass spectrometric conditions were as follows: spray voltage of 1.8 kV; capillary temperature of 180 °C; normalized collision energy of 35% at an activation of *q*=0.25 and an activation time of 30 ms. The LTQ-Orbitrap XL was operated in data-dependent mode. Survey full scan MS spectra were acquired in the orbitrap (*m*/*z* 300–2,000) with a resolution of 30,000 at *m*/*z* 400 and an automatic gain control target at 10^6^. The five most intense ions were selected for CID MS/MS fragmentation in the linear ion trap at an automatic gain control target of 30,000. Detection in the linear ion trap of previously selected ions was dynamically excluded for 30 s. Singly charged ions as well as ions with unrecognized charge state were also excluded. Internal calibration of the Orbitrap was performed using the lock mass option (lock mass: *m*/*z* 445.120025 (ref. 49)).

### Protein–protein cross-linking

Ten microlitres of a 1:1 mixture of 2.5 mM deuterated (d4) and non-deuterated (d0) BS3 (Thermo Scientific) were added to 240 μl of purified eIF2–eIF2B complex. The reaction mixture was incubated for 30 min at 26 °C and 450 r.p.m. in a thermomixer. One hundred and sixty microlitres of 100 mM ammonium bicarbonate were added to quench the remaining cross-linking reagent. The proteins were precipitated with ethanol and digested in solution with RapiGest SF Surfactant (see above) or separated by SDS–PAGE and digested in gel (see above). The peptides obtained from in-solution digestion were re-dissolved in 20% (v/v) ACN, 4% (v/v) FA and separated by cation exchange chromatography using SCX stage tips (Thermo Scientific) according to the manufacturer’s protocol. Peptides were eluted with different concentrations of ammonium acetate (25 mM–500 mM) and dried in a vacuum centrifuge. The mixture of cross-linked and non-cross-linked peptides was analysed by nanoLC-MS/MS.

Potential crosslinks were identified by using the MassMatrix Database Search Engine^50^,^51^,^52^. Search parameters were as follows: peptides were defined as tryptic with a maximum of two missed cleavage sites. Carbamidomethylation of cysteines and oxidation of methionine residues were allowed as variable modifications. The mass accuracy filter was 10 p.p.m. for precursor ions and 0.8 Da for fragment ions. Minimum pp and pp2 values were 5.0 and minimum pp_tag_ was 1.3. Maximum number of crosslinks per peptide was 1. All searches were performed twice, including the deuterated and the non-deuterated cross-linker, respectively. Potential crosslinks were validated manually by (i) checking the presence of the according peak pair in the MS spectra generated by the d4/d0-BS3 mixture and (ii) by the quality of the MS/MS spectrum.

### Absolute quantification of the eIF2–eIF2B complex

The proteins were digested with RapiGest and analysed by nanoLC-MS/MS (see above). The raw data were searched against Uniprot_yeast database using MaxQuant software^53^ v1.2.2.5. The mass accuracy filter was 20 p.p.m. for precursor ions and 0.5 Da for MS/MS fragment ions. Peptides were defined to be tryptic with maximal two missed cleavage sites. Carbamidomethylation of cysteines and oxidation of methionine residues were allowed as variable modifications. iBAQ^54^ was implemented in the MaxQuant search. Obtained iBAQ intensities of the eIF2–eIF2B proteins were compared to determine the stoichiometry of the two complexes.

### GTP binding

To investigate GTP binding, purified εγ-core complex was incubated with excess (10 mM) of 6-Thio-GTP. Ultraviolet-cross-linking was performed on ice at 365 nm for 5 min. The protein complex was analysed before (control) and after ultraviolet cross-linking as described above.

### DEPC labelling

Approximately 5 μg of eIF2B complex were incubated with 70 μM DEPC at 37 °C. After 1 min, the reaction was quenched by addition of 5 μl of 10 mM Imidazole. The proteins were digested with 0.4 μg Tryspin at 37 °C overnight and the peptides were subsequently subjected to LC-MS/MS analysis (see above). Modified sites were identified by database search using MassMatrix Database Search Engine (see above), allowing for DEPC modification at His, Tyr, Ser and Thr residues^55^.

### Homology modelling

Homology models for yeast eIF2 (α, β, γ) and eIF2B (α, β, δ, γ, ε) were generated using MODELLER web server (https://modbase.compbio.ucsf.edu/scgi/modweb.cgi) and SWISS-MODEL automated protein modelling server (http://swissmodel.expasy.org). eIF2 subunits (α res. 7–267, γ res. 96–527 and *β* res. 127–261) were modelled using the crystal structure of archaeal aIF2 from *S. solfataricus*, PDB 3CW2 as a template (Fig. 3a). Homologous regulatory subunits of eIF2B α and *β* were modelled based on a crystal structure of human eIF2Bα (PDB 3ECS) (SWISS-MODEL). For the δ-subunit, the best homologous structure based on the crystal structure of 5-methylthioribose 1-phosphate isomerase from *Bacillus subtilis* (PDB 2YVKA) (MODELLER) contained residues 245–536 within the homologous region. As most of the cross-links were identified within the C and N termini, we submitted the CTD (540–651) and NTD (1–244) sequences of the δ-subunit for homology modelling (MODELLER) separately. For these, we obtained homologous structures based on the crystal structure of Ribose-1,5-bisphosphate isomerase, *Thermococcus kodakaraensis* (PDB 3A11A) and (1YA9A) for CTD and NTD, respectively. The CTD structure constitutes part of the RFL domain, while the NTD corresponds mainly to the NαH domain (Fig. 4c).

We used AGP and GlmU as templates to obtain models of eIF2B γ- and ε-subunits. For eIF2Bγ, only the structure based on GlmU template reached completion when modelled (SWISS-MODEL). Therefore, we also modelled PL (44–314) and LβH (358- 578) domains of eIF2Bγ separately. Intra-subunit cross-links identified in this study within eIF2Bγ indicate that the arrangement of the PL and LβH domains is more consistent with the AGP-like rather than GlmU domain arrangement in agreement with previous experimental results^27^. In AGP, the PLD and LβH domains have more extensive interactions forming a more compact structure as opposed to the LβH domain extending away from the PL domain in GlmU. The eIF2Bε residues 1–520 excluding ε-cat were submitted for modelling to MODELLER. This resulted in a homology model of ε (residues 30–470) based on the AGP template (PDB 1YP2). For ε-cat, we used crystal structure available (PDB 1PAQ) for the yeast catalytic domain^18^.

## Additional information

**How to cite this article:** Gordiyenko, Y. *et al*. eIF2B is a decameric guanine nucleotide exchange factor with a γ_2_ε_2_ tetrameric core. *Nat. Commun.* 5:3902 doi: 10.1038/ncomms4902 (2014).

## Supplementary Material

Supplementary Information Supplementary Figures 1-5 and Supplementary Tables 1-6
