# Supplementary material for: eIF2B is a decameric guanine nucleotide exchange factor with a γ2ε2 tetrameric core
Source: Nat Commun. 2014 May 23;5:3902. doi: 10.1038/ncomms4902 (PMC4046112; doi:10.1038/ncomms4902)
Supplement: Supplementary Information — Supplementary Figures 1-5 and Supplementary Tables 1-6 [file ncomms4902-s1.pdf]

Figures

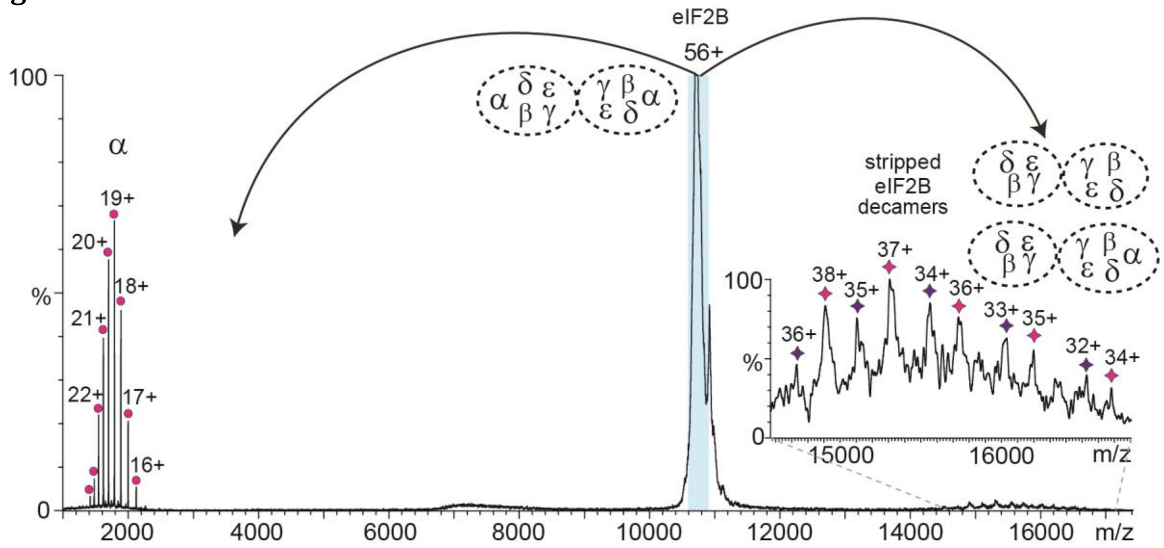

Supplementary Figure 1. Tandem MS of yeast eIF2B.

Tandem MS of the 56+ charge state (shaded blue) of the eIF2B dimer showing that two α subunits (● at ~ 2000 m/z) dissociate from the dimer when collision energy is increased resulting in the formation of the corresponding stripped complexes corresponding to the loss of one (✦ at 15000 - 16000 m/z) or two (✦ at 15000 - 16000 m/z) α subunits and indicating peripheral location of the α subunit in the complex. The spectrum represents an experiment from at least three biological replicates.

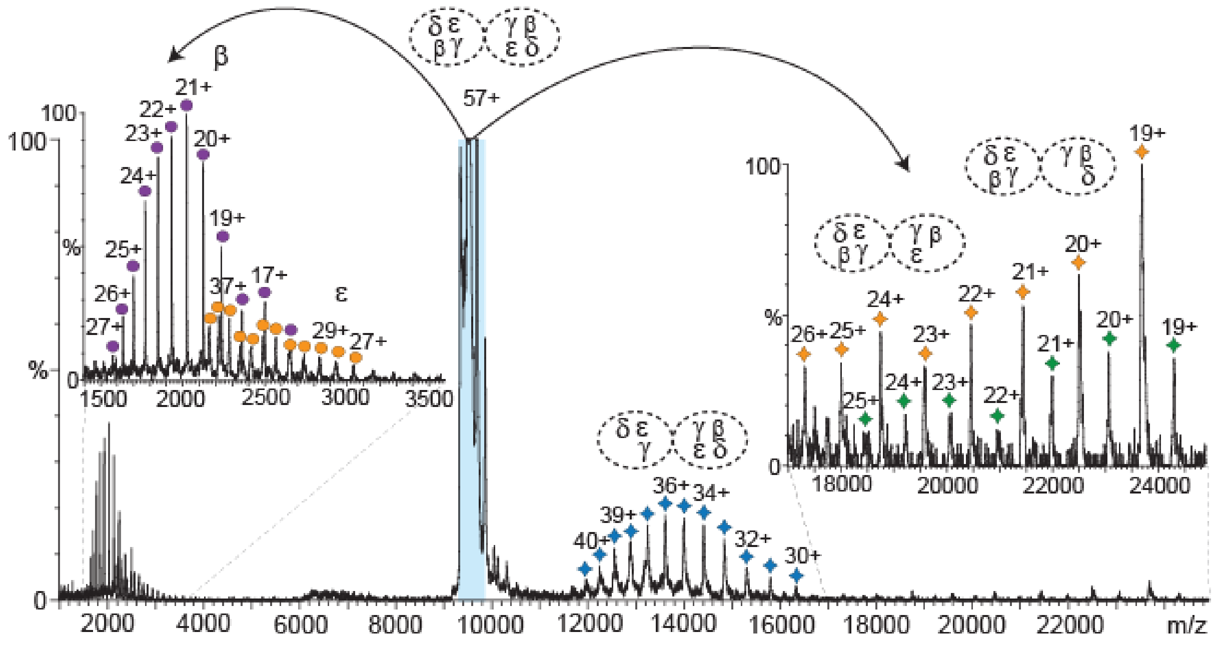

Supplementary Figure 2. Tandem MS of yeast eIF2Bβδγε sub-complex.

Tandem MS of the 57+ charge state (shaded blue) of the eIF2Bβδγε octamer showing β subunits (● at 1500-2500 m/z) dissociating most readily from the complex under increased collision energy which resulted in the formation of the corresponding β-stripped complexes (✦ at 12000 - 16000 m/z). Much lower intensity species were observed for the ε subunit (● at 2200-3000 m/z) dissociated from the complex and corresponding stripped complexes (✦ at 18000-24000 m/z). Stripped complexes lacking δ subunit were also observed (✦ at 18000-24000 m/z) at very low intensity, however no peaks could be assigned for the dissociated δ subunit. The spectrum represents an experiment from two biological replicates.

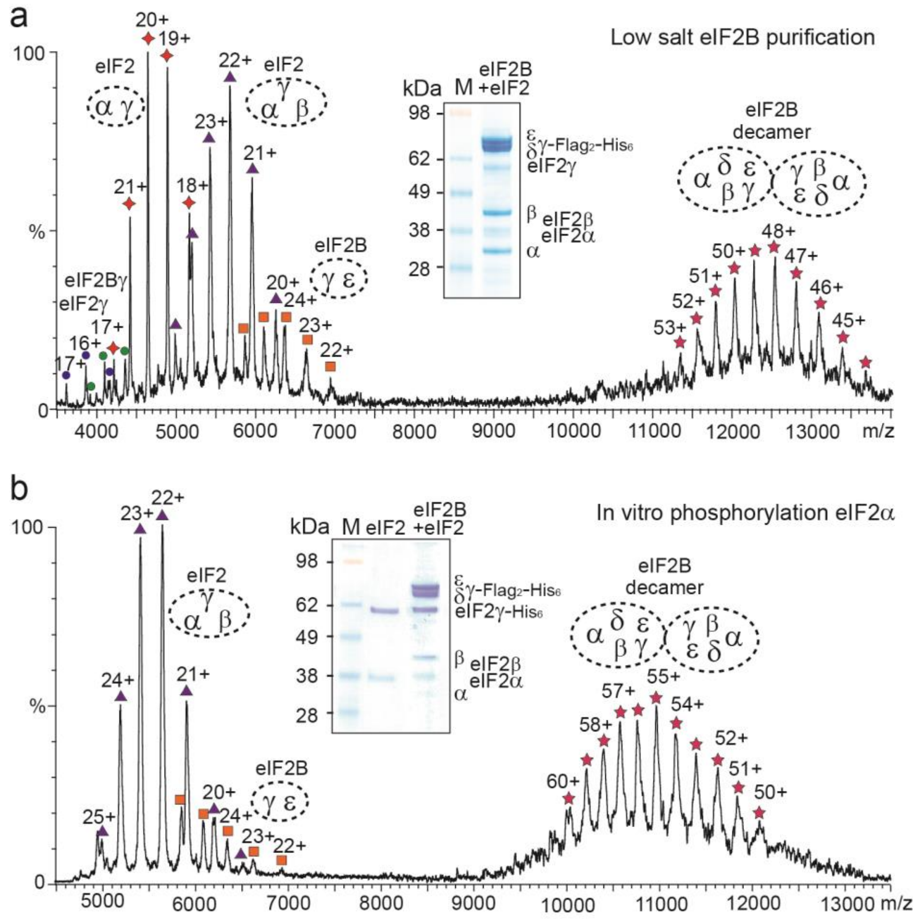

Supplementary Figure 3. The interaction between eIF2 and eIF2B is transient.

a - SDS-PAGE of the eIF2-eIF2B complex purified from yeast through the FLAG-tag on the γ subunit of eIF2B at low salt conditions (insert), showing that interaction between eIF2 and eIF2B can be maintained during purification with sub-stoichiometric amounts of eIF2 relative to eIF2B, and native MS of the low salt purified eIF2-eIF2B complex showing separate peaks for the eIF2B (★ at 11000 - 13000 m/z) and eIF2 (▲ at 5000 - 6000 m/z) complexes. Additional species are present corresponding in mass to eIF2 α/γ dimer (✦ at 4500 - 5000 m/z), eIF2γ (● at 4000 m/z), eIF2Bγ (● at 4000 m/z) and eIF2Bε/δ subcomplex (■ at 6000 - 7000 m/z).

b - SDS-PAGE (insert) and native MS of the eIF2-eIF2B complex assembled on anti-FLAG sepharose attached eIF2B pre-washed in a high salt buffer to disrupt the interactions with endogenous eIF2 and purified in vitro phosphorylated eIF2. While increased amount of eIF2 retained in the complex with eIF2B after phosphorylation at Ser51 (compare to Supplementary Fig. S3a insert), native MS showed separate species for eIF2B (★ at 10000-12000 m/z) and eIF2 (▲ at 5000-6000 m/z) indicating their transient interaction. Low intensity species corresponding to eIF2Bγε subcomplex were present (■ at 6000-7000 m/z).

The spectra shown represent an experiment from at least three biological replicates.

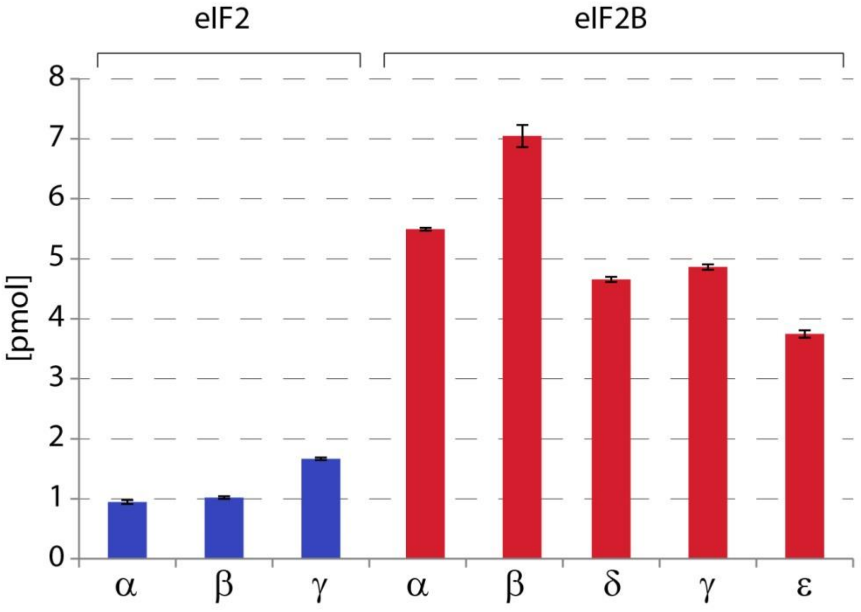

Supplementary Figure 4. Label-free absolute quantification (iBAQ) of eIF2:eIF2B complex.

Absolute quantities are given in pmol for eIF2 (blue bars) and eIF2B (red bars) subunits. Error bars show standard deviation determined from three technical replicates are given.

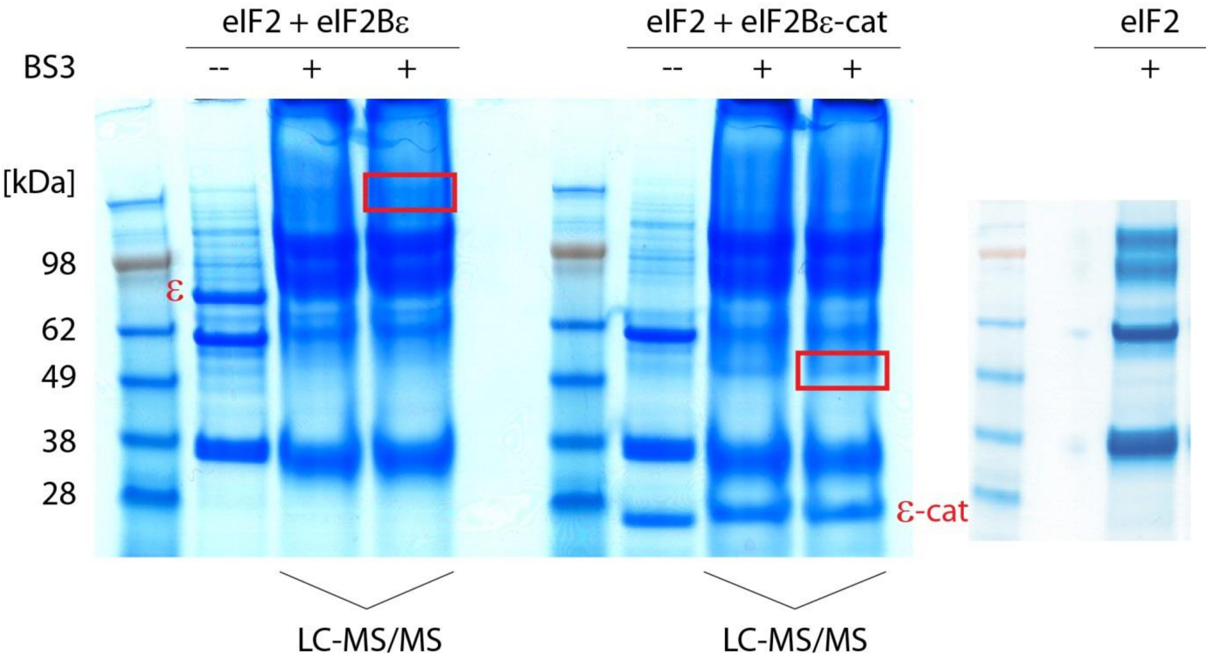

Supplementary Figure 5. Cross-linking of eIF2 and eIF2Bε and catalytic domain of eIF2Bε.

eIF2 was incubated with full-length eIF2Bε and the catalytic domain of eIF2Bε in the absence (--) and presence (+) of BS3-cross-linker. Additional protein bands after cross-linking confirm interactions between eIF2 and eIF2Bε. Cross-linking of eIF2 is shown as a control.

Supplementary Table 1. Masses of proteins and protein complexes identified in eIF2 and eIF2B complexes.

**Protein/complex**	**Theoretical mass, Da**	**Calculated mass of the complex based on measured masses of individual proteins, Da**	**Measured mass, Da**
eIF2α	34718	N/A	34799.09 ± 8.80
eIF2β	31574	N/A	30894.06± 9.22
eIF2γ	57866	N/A	57883.77 ± 25.32
eIF2γ-His$_6$	58707	N/A	58919.73 ± 23.77
eIF2γ-His$_6$ GDP	59150	N/A	59338.20 ± 22.11
eIF2α/γ	92584	92663	92634.34 ± 15.25
eIF2α/γ-His$_6$	93425	93719	93800.80± 24.45
eIF2α/γ-His$_6$+GDP	93868	94162	94236.27± 22.15
eIF2α/β/γ	124158	124237	124654.16 ± 32.24
eIF2α/β/γ-His$_6$	124999	124613	124809.35± 21.86
eIF2Bα	34025	N/A	33938.35 ± 0.57
eIF2Bβ	42570	N/A	42481.09 ± 5.72
eIF2Bγ-Fl$_2$-His$_6$	69376	N/A	69399.73 ± 11.37
eIF2Bε	81161	N/A	82096.53 ± 25.96
eIF2Bε-Fl	81161	N/A	85889 ± 8.46
eIF2Bγ/ε	150537	151497	152537.98 ± 51.87
eIF2Bγ-Fl/ε (core)	150537	151845	152544.35 ± 40.38
eIF2B(γ/ε) x 2	301074	302994	305122.28 ± 48.22
eIF2B(γ-Fl/ε) x 2 (core)	301074	303532	306362 ± n/a
eIF2B(βδγε)x2	527918	529660	532230.50 ± 35.72
eIF2B(βδγε)x2 - β	485348	487179	489650.22 ± 55.90
eIF2B(βδγε)x2 - ε	446757	447563	449863.25 ± 50.34
eIF2B(βδγε)x2 - δ	457066	458808	461244.63 ± 139.52
eIF2B(αβδγε)x2	595968	597536	600893.81 ± 49.52
eIF2B(αβδγε)x2 - α	561943	563598	566299.19 ± 59.85
eIF2B(αβδγε)x2 - 2α	527918	529660	528510.38 ± 40.13

Supplementary Table 2. Intra-cross-links in eIF2B subunits.

Residue 1	Residue 2	Complex	Distance C_α–C_α (Å)	Residue 1	Residue 2	Complex	Distance C_α–C_α (Å)
**α subunit intra**				**δ subunit intra**			
α Lys107	α Lys145	2+2B	14.77	δ Lys 7	δ Lys 365	2+2B	-
α Lys107	α Lys111	2+2B	6.34	δ Lys 33	δ Lys 40	2Bβδγε	-
**β subunit intra**				δ Lys 33	δ Lys 43	2+2B	-
β Lys136	β Lys147	2Bβδγε	20.15	δ Lys 40	δ Lys 47	2Bβδγε	10.20
β Lys145	β Lys147	2Bβδγε	7.27	δ Lys 40	δ Lys 96	2+2B	32.44
β Lys224	β Lys231	2Bβδγε	10.18	δ Lys 43	δ Lys 50	2+2B	14.34
β Lys378	β Lys380	2Bβδγε	-	δ Lys 47	δ Lys 133	2+2B	-
**γ subunit intra**				δ Lys 72	δ Lys 575	2Bβδγε	-
γ Lys 147	γ Lys 157	2Bβδγε	12.33	δ Lys 73	δ Lys 76	2+2B/2Bβδγε	2.39
γ Lys 211	γ Lys 212	2Bβδγε/ 2Bγε	2.53	δ Lys 76	δ Lys 85	2Bβδγε	14.08
γ Lys 211	γ Lys 216	2Bβδγε/ 2Bγε	5.66	δ Lys 85	δ Lys 89	2Bβδγε	5.99
γ Lys 211	γ Lys 410	2Bβδγε/ 2Bγε	-	δ Lys 89	δ Lys 133	2Bβδγε	-
γ Lys 249	γ Lys 251	2Bβδγε/ 2Bγε	-	δ Lys 95	δ Lys 133	2+2B/2Bβδγε	-
γ Lys 412	γ Lys 419	2Bβδγε	19.23	δ Lys 97	δ Lys 104	2Bβδγε	15.30
γ Lys 345	γ Lys 376	2Bγε	-	δ Lys 97	δ Lys 133	2+2B	-
γ Lys 345	γ Lys 346	2Bγε	-	δ Lys 97	δ Lys 556	2+2B	-
γ Lys 116	γ Lys 140	2+2B/2Bγε	-	δ Lys 133	δ Lys 559	2+2B	-
γ Lys 221	γ Lys 410	2+2B	-	δ Lys 133	δ Lys 562	2+2B	-
γ Lys 249	γ Lys 362	2+2B/2Bβδγε / 2Bγε	-	δ Lys 133	δ Lys 570	2Bβδγε	-
γ Lys 251	γ Lys 362	2+2B	-	δ Lys 332	δ Lys 572	2Bβδγε	-
γ Lys 410	γ Lys 412	2+2B	-	δ Lys 547	δ Lys 559	2+2B/2Bβδγε	20.93
γ Lys 410	γ Lys 494	2+2B	-	δ Lys 547	δ Lys 562	2+2B/2Bβδγε	20.75
**ε subunit intra**				δ Lys 556	δ Lys 559	2+2B/2Bβδγε	5.14
ε Lys 4	ε Lys 9	2+2B/2Bβδγε	-	δ Lys 556	δ Lys 562	2+2B/2Bβδγε	7.54
ε Lys 5	ε Lys 9	2+2B	-	δ Lys 556	δ Lys 579	2+2B	18.62
ε Lys 8	ε Lys 9	2+2B/2Bβδγε	-	δ Lys 559	δ Lys 570	2Bβδγε	28.52
ε Lys 4	ε Lys 520	2+2B/2Bγε	-	δ Lys 559	δ Lys 579	2+2B	20.23
ε Lys 9	ε Lys 17	2Bβδγε/ 2Bγε	-	δ Lys 562	δ Lys 580	2+2B	22.56
ε Lys 176	ε Lys 189	2Bγε	25.13	δ Lys 570	δ Lys 575	2Bβδγε	12.15
ε Lys 327	ε Lys 329	2Bγε	6.71	δ Lys 570	δ Lys 579	2Bβδγε	19.94
ε Lys 338	ε Lys 356	2+2B/2Bβδγε / 2Bγε	16.56	δ Lys 570	δ Lys 580	2Bβδγε	3.85
ε Lys 431	ε Lys 438	2Bβδγε/ 2Bγε / 2Bε	-	δ Lys 572	δ Lys 575	2Bβδγε	10.20
ε Lys 690	ε Lys 691	2Bβδγε/ 2Bγε / 2Bε-cat	3.82	δ Lys 575	δ Lys 580	2+2B/2Bβδγε	16.88
				δ Lys 579	δ Lys 587	2Bβδγε	7.96

Supplementary Table 3. Inter-cross-links between eIF2B subunits.

Residue 1	Residue 2	Complex
**2Bα - 2Bγ inter**		
α Lys 107	γ Lys 412	2+2B
**2Bβ - 2Bδ inter**		
β Lys 136	δ Lys 648	2Bβδγε
β Lys 380	δ Lys 95	2+2B
β Lys 380	δ Lys 570	2Bβδγε
**2Bβ - 2Bγ inter**		
β Lys 329	γ Lys 494	2Bβδγε
**2Bβ - 2Bε inter**		
β Lys 380	ε Lys 9	2+2B
**2Bγ -2Bδ inter**		
γ Lys 249	δ Lys 76	2+2B
γ Lys 410	δ Lys 422	2+2B
γ Lys 410	δ Lys 559	2+2B
γ Lys 410	δ Lys 562	2+2B
γ Lys 410	δ Lys 570	2Bβδγε
γ Lys 410	δ Lys 575	2+2B
γ Lys 410	δ Lys 579	2+2B
γ Lys 410	δ Lys 580	2+2B
γ Lys 412	δ Lys 133	2Bβδγε
**2Bγ - 2Bε inter**		
γ Lys 376	ε Lys 176	2+2B
γ Lys 249	ε Lys 176	2Bγε (Tri)
γ Lys 249	ε Lys 517	2Bβδγε
γ Lys 249	ε Lys 519	2Bγε (Tri)
**2Bδ - 2Bε inter**		
δ Lys 556	ε Lys 4	2+2B
δ Lys 564	ε Lys 4	2+2B
δ Lys 421	ε Lys 356	2+2B

Supplementary Table 4. eIF2 intra- and inter- cross-links.

Residue1	Residue 2	Distance $C_\alpha - C_\alpha$ (Å)	Complex	Residue 1	Residue 2	Distance $C_\alpha - C_\alpha$ (Å)	Complex
**2α subunit intra**							
α Lys 61	α Lys 87	14.49	2-2B/2-2Bε/2-2Bε-cat	α Lys 78	α Lys 80	6.20	2-2Bε/2-2Bε-cat
α Lys 118	α Lys 175	14.58	2-2B	α Lys 106	α Lys 183	23.98	2-2Bε
α Lys 67	α Lys 61	10.57	2-2Bε/2-2Bε-cat	α Lys 118	α Lys 175	14.58	2-2Bε
α Lys 67	α Lys 78	27.04	2-2Bε/2-2Bε-cat	α Lys 104	α Lys 106	5.47	2-2Bε/2-2Bε-cat
α Lys 67	α Lys 87	13.30	2-2Bε/2-2Bε-cat	α Lys 170	α Lys 175	8.38	2-2Bε
α Lys 67	α Lys 106	15.98	2-2Bε/2-2Bε-cat	α Lys 221	α Lys 175	26.21	2-2Bε
α Lys 67	α Lys 183	34.89	2-2Bε	α Lys 221	α Lys 183	14.62	2-2Bε-cat
α Lys 78	α Lys 87	22.08	2-2Bε	α Lys 239	α Lys 175	19.92	2-2Bε-cat
**2β subunit intra**							
β Lys 82	β Lys 83	-	2-2Bε	β Lys 170	β Lys 216	11.24	2-2Bε
β Lys 87	β Lys 89	-	2-2Bε/2-2Bε-cat	β Lys 171	β Lys 214	6.75	2-2Bε/2-2Bε-cat
β Lys 89	β Lys 98	-	2-2Bε	β Lys 214	β Lys 281	-	2-2Bε/2-2Bε-cat
β Lys 157	β Lys 240	10.55	2-2B	β Lys 214	β Lys 216	5.27	2-2Bε/2-2Bε-cat
β Lys 157	β Lys 281	-	2-2Bε	β Lys 216	β Lys 281	-	2-2Bε
β Lys 214	β Lys 281	-	2-2B	β Lys 216	β Lys 220	12.73	2-2Bε
β Lys 170	β Lys 171	3.79	2-2Bε/2-2Bε-cat	β Lys 272	β Lys 281	-	2-2Bε
β Lys 170	β Lys 214	8.40	2-2Bε/2-2Bε-cat				
**2γ subunit intra**							
γ Lys 498	γ Lys 518	9.99	2-2B/2-2Bε	γ Lys 455	γ Lys 479	11.68	2-2Bε
γ Lys 165	γ Lys 113	16.39	2-2B	γ Lys 479	γ Lys 518	12.41	2-2Bε/2-2Bε-cat
γ Lys 264	γ Lys 268	6.15	2-2B/2-2Bε	γ Lys 479	γ Lys 519	14.35	2-2Bε/2-2Bε-cat

γ Lys 129	γ Lys 507	28.40	2-2Bε/2-2Bε-cat	γ Lys 165	γ Lys 168	6.33	2-2Bε/2-2Bε-cat
γ Lys 129	γ Lys 139	15.19	2-2Bε/2-2Bε-cat	γ Lys 265	γ Lys 269	6.15	2-2Bε-cat
γ Lys 129	γ Lys 325	17.50	2-2Bε	γ Lys 325	γ Lys 334	9.98	2-2Bε
γ Lys 139	γ Lys 507	18.67	2-2Bε	γ Lys 362	γ Lys 365	9.59	2-2Bε/2-2Bε-cat
γ Lys 444	γ Lys 452	11.37	2-2Bε	γ Lys 444	γ Lys 449	4.83	2-2Bε/2-2Bε-cat
γ Lys 455	γ Lys 452	9.30	2-2Bε	γ Lys 449	γ Lys 452	8.08	2-2Bε/2-2Bε-cat
**2α-β inter**							
α Lys 61	β Lys 170		2-2Bε	α Lys 61	β Lys 272		2-2Bε
α Lys 61	β Lys 214		2-2Bε	α Lys 61	β Lys 281		2-2Bε/2-2Bε-cat
**2α-γ inter**							
α Lys 61	γ Lys 325		2-2Bε/2-2Bε-cat	α Lys 80	γ Lys 268		2-2Bε-cat
α Lys 61	γ Lys 129		2-2Bε-cat	α Lys 183	γ Lys 452		2-2Bε
α Lys 61	γ Lys 165		2-2Bε-cat				
**2γ-β inter**							
γ Lys 184	β Lys 19/86[1]		2-2B	γ Lys 268	β Lys 102		2-2Bε
γ Lys 449	β Lys 260		2-2B	γ Lys 129	β Lys 157		2-2Bε
γ Lys 401	β Lys 89		2-2B	γ Lys 165	β Lys 54		2-2Bε-cat

[1] Not unique, either residue might be cross-linked 2β Lys 19 or 2β Lys 86

Supplementary Table 5. Cross-links between eIF2 and eIF2B.

Residue1 eIF2	Residue 2 eIF2B	Complex
2α Lys 67	Bε Lys 691	2-2B
2α Lys 175	Bδ Lys 365	2-2B
2β Lys 170	Bα Lys 145	2-2B
2β Lys 240	Bγ Lys 249	2-2B
2β Lys 183	Bγ Lys 249	2-2B
2β Lys 247	Bε Lys 17	2-2Bε
2β Lys 260	Bε Lys 154	2-2Bε
2β Lys 19/86[1]	Bδ Lys 439	2-2B
2β Lys 247	Bδ Lys 422	2-2B
2γ Lys 66	Bγ Lys 249	2-2B
2γ Lys 113	Bγ Lys 249	2-2B
2γ Lys 53	Bδ Lys 32	2-2B
2γ Lys 518	Bδ Lys 89	2-2B

Supplementary Table 6. Homology models of eIF2 and eIF2B subunits.

Segment submitted	Modelling server	Modeled segment	Model score	PDB template code	Sequence identity (%)	PDB comment
eIF2α 1-304	SWISS-MODEL	7-267	0.55	3CW2C	19.6	aIF2α, *Sulfolobus solfataricus*
eIF2β 1-285	SWISS-MODEL	127-261	0.27	3CW2K	30.4	aIF2β, *Sulfolobus solfataricus*
eIF2γ 1-527	MODELLER	96-519	1.0	1KK1A	48	eIF2γ, *Pyrococcus abyssi*
eIF2Bα 1-305	SWISS-MODEL	1-304	1.0	3ECSA	44	Human eIF2Bα
eIF2Bβ 1-381	SWISS-MODEL	60-371	1.0	3ECSA	23	Human eIF2Bα
eIF2Bδ 1-651	MODELLER	245-536	1.0	2YVKA	18	5-methylthioribose 1-phosphate isomerase, *Bacillus subtilis*
eIF2Bδ 1-244	MODELLER	36-105	0.03	1YA9A	13	Mouse apolipoprotein E
eIF2Bδ 538-651	MODELLER	540-651	0.21	3A11A	21	Ribose-1,5-bisphosphate isomerase, *Thermococcus kodakaraensis*
eIF2Bγ	SWISS-MODEL	44-314	0.33	1YP2A	10.1	Potato tuber ADP-glucose pyrophosphorylase
eIF2Bγ		375-565	0.3	2OI7A	10.4	GlmU acetyltransferase, *E. coli*
eIF2Bε 1-520	SWISS-MODEL	30- 431	0.27	1YP2A	14.9	Potato tuber ADP-glucose pyrophosphorylase
